# Metabolic dysfunction-associated fatty liver disease as a mediator of urolithiasis risk: evidence from cross-sectional and genetic studies

**DOI:** 10.7150/ijms.106824

**Published:** 2025-01-21

**Authors:** Lin-Tao Miao, Wen-Jie Wang, Jin-Zhou Xu, Ming-Liang Zhong, Yuan Gong, Cong Li, Yang Xun, Shao-Gang Wang

**Affiliations:** 1 Department of Urology, Tongji Hospital, Tongji Medical College, Huazhong University of Science and Technology, Wuhan, China.; 2 Health Management Center, Tongji Medical College, Tongji Hospital, Huazhong University of Science and Technology, Wuhan, China.; These authors contributed equally to this work: Lin-Tao Miao and Wen-Jie Wang.

**Keywords:** urolithiasis, metabolic dysfunction-associated fatty liver disease, nonalcoholic fatty liver disease, cross-sectional study, Mendelian randomization, Mediation analysis

## Abstract

**Introduction:** The rising prevalence of urolithiasis and metabolic dysfunction-associated fatty liver disease (MAFLD) has become a significant concern within urology and hepatology, respectively. Emerging studies reveal a compelling association between these conditions, yet the underlying relationship remains poorly understood. This study aims to investigate the connection between urolithiasis and MAFLD within the Chinese population and leverages Mendelian Randomization (MR) analysis to explore potential causal links between the two diseases, shedding light on new avenues for both prevention and treatment.

**Methods:** This cross-sectional study included 98,232 Chinese participants and employed logistic regression models and subgroup analyses to assess the association between MAFLD and urolithiasis. For the MR analysis, genetic instruments from genome-wide association studies served as instrumental variables. Bidirectional MR was conducted to investigate the potential causal relationship between genetically predicted MAFLD and urolithiasis. Additionally, multivariable MR and mediation analysis were used to assess both the direct effect of MAFLD on urolithiasis and any mediating pathways involved.

**Results:** In a cohort of 98,232 Chinese participants, 10.1% (9,928) had urolithiasis, and 26.7% (26,217) had MAFLD. MAFLD was positively associated with urolithiasis, with an unadjusted odds ratio (OR) of 1.563 (95% CI, 1.495-1.633), an adjusted OR in model 1 of 1.204 (95% CI, 1.146-1.265), and an adjusted OR in model 2 of 1.137 (95% CI, 1.079-1.199). Subgroup analysis showed consistent associations across most subgroups, except for a significant interaction between MAFLD and triglyceride (TG) levels (p for interaction < 0.05). Bidirectional MR analysis suggested that genetically predicted MAFLD increased the risk of urinary stone disease, while no significant causal effect was observed from urolithiasis to MAFLD. Furthermore, multivariable MR and mediation analyses highlighted MAFLD as a key mediator in kidney stone formation driven by obesity and type 2 diabetes.

**Conclusions:** This study demonstrates a causal link between MAFLD and an increased risk of urolithiasis, supported by both epidemiological and genetic evidence. Furthermore, MAFLD serves as a significant mediator in the pathway from obesity and type 2 diabetes to urolithiasis development.

## Introduction

Urolithiasis is an increasingly common, costly, and recurrent condition in urology, causing significant suffering for patients and imposing a financial burden on healthcare systems. With the rise in living standards and advancements in imaging technologies, particularly computed tomography (CT), the incidence of urolithiasis has surged. From 1990 to 2019, the global incidence of urolithiasis increased by 48.57% over three decades^1^. Current estimates place the prevalence of kidney stones at 4% in South America, 1-19% in Asia, and 5-10% in Europe^2^. In China, a recent cross-sectional study found that approximately 1 in 17 adults has kidney stones^3^. The financial burden has also grown significantly, with the annual cost in the U.S. alone reaching an estimated $5.3 billion in 2014^4^. Given these trends, it is critical to study the factors influencing urolithiasis to develop targeted prevention and treatment strategies.

Metabolic dysfunction-associated fatty liver disease (MAFLD) was introduced in 2020 by international hepatology experts as a new definition intended to replace non-alcoholic fatty liver disease (NAFLD)^5^. MAFLD is the most typical chronic liver condition, and its global prevalence has reached 25.24%^6^. MAFLD is associated with numerous extrahepatic comorbidities, among which urolithiasis is a significant one^7^, ^8^. In addition, several investigations have demonstrated a strong link between urolithiasis and MAFLD. A cross-sectional study from southern China suggested that MAFLD may serve as a risk factor for urinary stones^9^. A recent systematic review and meta-analysis of seven studies involving 226,541 participants estimated that the risk of urolithiasis in MAFLD patients is 1.73 times higher than in healthy controls^10^. Although many studies have attempted to reveal the relationship between MAFLD and urolithiasis, it is difficult to infer the true causal relationship due to some confounding factors^10^-^14^. Mendelian randomization (MR) is a genetic approach that leverages data from large-scale genome-wide association studies (GWAS) to estimate disease risk^15^. MR studies employ genetic variants as instrumental variables to derive valid causal inferences, effectively minimizing the influence of confounding factors^16^.

Therefore, we first performed an analysis to verify the correlation between MAFLD and urolithiasis in the Chinese population and then explored the possible causal relationship between MAFLD and urolithiasis by a bidirectional MR study. Additionally, we employed multivariable MR and mediation analysis to investigate the mediating role of MAFLD, providing new insights into the diagnosis and treatment of urolithiasis.

## Methods

### Study population

**Figure 1:**
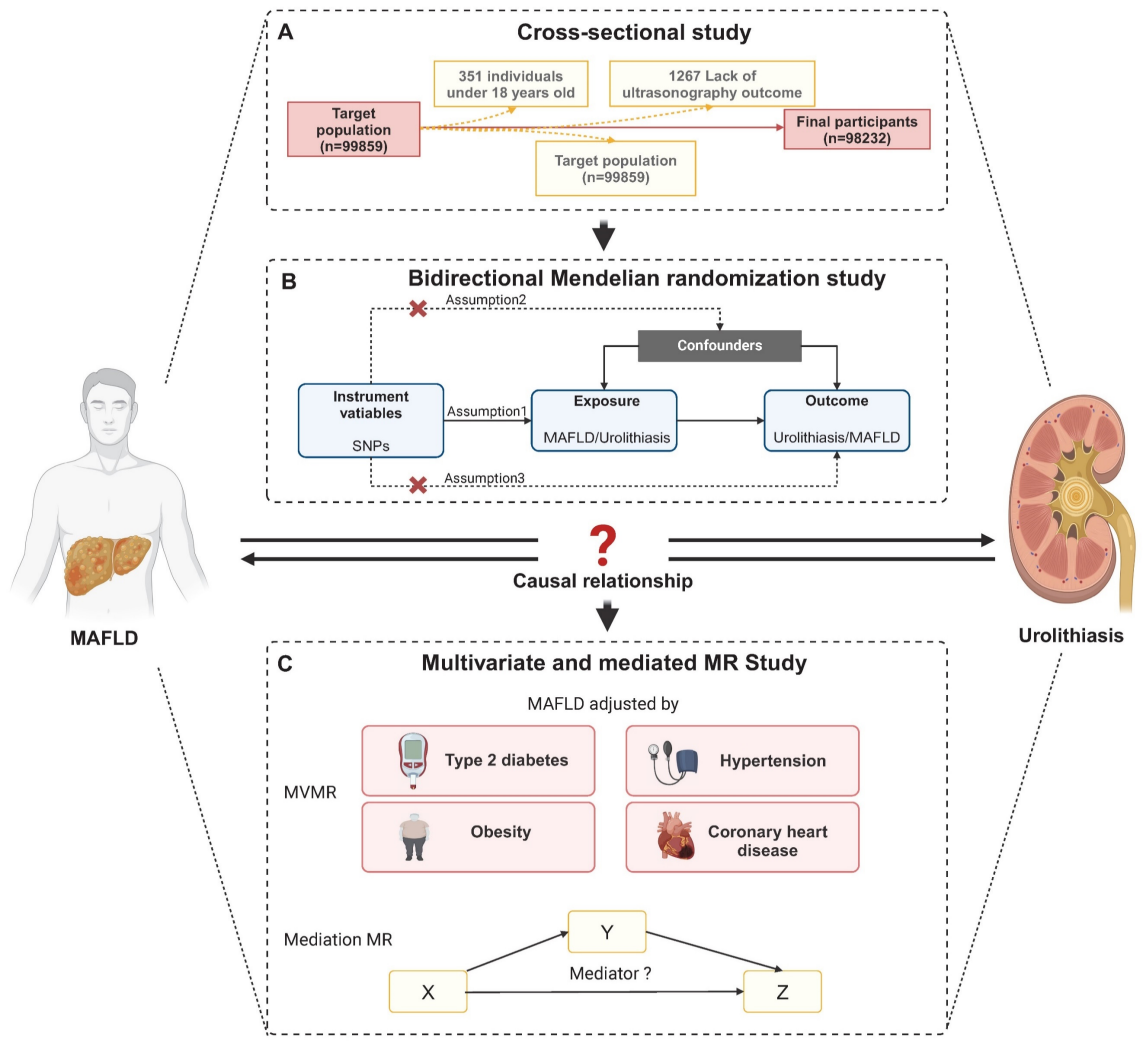
**Overview of Study Design and Analytical Framework. (A)** Flow chart of selecting participants in the study**.** After rejecting 1627 participants due to lack of ultrasonography outcome (n = 1267), age under 18, kidney deformity (n = 14), kidney transplantation (n = 23), or solitary kidney (n = 205), 98232 participants were then recruited based on their completion of a health examination. **(B)** Overview of bidirectional MR study**. (B)** Overview of multivariate and mediation MR study. Abbreviations: SNPs, single-nucleotide polymorphisms; MAFLD, metabolic dysfunction-associated fatty liver disease.

The study consisted of individuals who received a thorough medical examination at Tongji Hospital's Health Management Center in 2017. A total of 99,859 individuals' data and test results were collected. After rejecting 1627 participants due to age under 18, lack of ultrasonography outcome (n = 1267), kidney deformity (n = 14), kidney transplantation (n = 23), or solitary kidney (n = 205), 98232 participants were then recruited based on their completion of a health examination (**Figure 1A**). Tongji Hospital, Tongji Medical College, and Huazhong University of Science and Technology Institutional Review Boards gave their approval for our study (Approval ID: TJ- C20160115). This study complied with the Declaration of Helsinki's ethical principles. Each subject gave their informed consent.

### Measurement

Urolithiasis and MAFLD were diagnosed based on ultrasonography (US) examination. In addition to collecting data on demographics, comorbidities including obesity (BMI>24), high blood pressure (HBP), diabetes mellitus (DM), coronary heart disease (CHD), and MAFLD were also collected. Physical features including systolic blood pressure (SBP), diastolic blood pressure (DBP), and body mass index (BMI, which is calculated by dividing one person's weight in kilograms by their height in square meters) were collected as routine data for medical examinations. Laboratory indicators, including alanine aminotransferase (ALT), aspartate aminotransferase (AST), total protein (TP), albumin (Alb), globulin (Glo), -glutamyl transpeptidase (GGT), total bilirubin (TBIL) indirect bilirubin (IBIL), direct bilirubin (DBIL), total cholesterol (TC), high-density lipoprotein cholesterol (HDL), low-density lipoprotein cholesterol (LDL), triglycerides (TG), serum creatinine (SCr), uric acid (UA), fasting glucose (Glu), and blood platelet(PLT) were measured by blood specimens collected from the anterior vein of the elbow. Urine pH (UpH) was acquired from a urinalysis, which can indicate the crystal type of kidney stones^17^. The estimated glomerular filtration rate (eGFR) was calculated using the CKD-EPI China equation, which includes a correction factor of 1.1 for the Chinese population. In this equation, the coefficient κ is 0.7 for women and 0.9 for men, while α is set at -0.329 for women and -0.411 for men. The term "min" represents the minimum value between SCr/κ and 1, while "max" indicates the maximum value between SCr/κ and 1^18^:



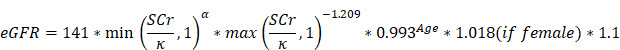



### Statistical analysis

Data are presented as mean ± standard deviation (SD) for continuous normally distributed variables or as counts with percentages for categorical variables. Logistic regression models were employed to assess the association between MAFLD and urolithiasis. Models were sequentially adjusted for age, sex, and clinical characteristics, including obesity (absent/present), HBP (absent/present), DM (absent/present), and CHD (absent/present) (model 1), plus biochemical indices including ALT, AST, Alb, Glo, HDL, LDL, TG, UA, Glu, UpH and eGFR (model 2).

A subgroup analysis was conducted using logistic regression (Model 2) to evaluate the odds ratio (OR) of urolithiasis in individuals with MAFLD compared to those without. Variables were categorized into the following groups: age (≤29, 30-44, 45-59, ≥60 years), sex (female/male), SBP (<140, ≥140 mmHg), DBP (<90, ≥90 mmHg), obesity (absent/present), HBP (absent/present), DM (absent/present), CHD (absent/present), ALT (<40, ≥40 mmol/L), AST(<40, ≥40 mmol/L), Alb (<35, ≥35 mmol/L), Glo (<25, ≥25 mmol/L), HDL (<1.0, ≥1.0 mmol/L), LDL (<3.4, ≥3.4 mol/L), TG (<1.7, ≥1.7 mmol/L), UA (for females <290 and for males <330 mmol/L; for females ≥290 and for males ≥330 mmol/L, according to our former research), Glu (<6.1, ≥6.1 mmol/L), eGFR (<90, 90~119, ≥120 mL/min/1.73 m^2^), UpH (<6.0, ≥6.0), PLT(<100, ≥100 mmol/L). The Wald test was used to test the interaction across subgroups.

### MR study

The flow chart of MR analysis and its three important assumptions is displayed in **Figure 1B**. The first presumption is that exposure should be substantially correlated with the genetic variants provided as instrumental variables (IVs); the second suggests that there should not be any confounding factors that could affect the exposure-outcome association; and the third is that the IVs should not affect the outcome unless by means of an association with the exposure.

We utilized data from the FinnGen database (R10) on MAFLD, urolithiasis, obesity, type 2 diabetes, hypertension, and coronary heart disease (https://www.finngen.fi/en), a unique study integrating genomic information with digital health records. Detailed information for each dataset is provided in **Table 1**. For MAFLD, 31 independent single-nucleotide polymorphisms (SNPs) (P < 1 × 10^-5^, r^2^ < 0.001, and clump distance >10,000 kb) were selected as genetic instrumental variables **(Supplementary Table 1)**, and 72 independent SNPs were used for urolithiasis **(Supplementary Table 2)**. The F-statistic was employed to assess the strength of each SNP instrument, and it was calculated using the following formula: F= R^2^ (N-2) /(1-R^2^), where R^2^ is the proportion of MAFLD variability explained by each instrument and N represents the sample size of the GWAS study. To determine the value of R^2^, we employed the following formula:



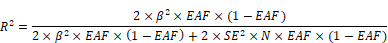



where EAF represents the effect allele frequency, beta denotes the estimated genetic effect on the exposure, N represents the sample size of the GWAS dataset, and SE stands for the standard error of the genetic effect^19^.

Mendelian randomization-Egger (MR Egger), weighted median regression, inverse variance weighting (IVW), simple mode, and weighed mode were the five MR methods used. The primary statistical model was the IVW method, and the random-effects method was utilized to assess the causal association. To ensure the robustness of the findings, several sensitivity analyses were performed. Cochran's Q test assessed heterogeneity, while the MR-Egger intercept checked for horizontal pleiotropy. Additionally, a leave-one-out (LOO) analysis was carried out to confirm that no single genetic variant disproportionately influenced the results. Finally, funnel plots were examined for potential small-study effects or publication bias. We performed multivariable MR analyses for urolithiasis, incorporating obesity, type 2 diabetes, hypertension, coronary heart disease, and MAFLD together as exposures. This analysis aimed to determine whether MAFLD has an independent causal effect on urolithiasis, separate from these established risk factors for stones. After adjusting for obesity and type 2 diabetes, the causal effect of MAFLD on urolithiasis disappeared. We hypothesize that MAFLD acts as a mediator in the formation of kidney stones induced by obesity and type 2 diabetes. To test this hypothesis, we conducted a two-step mediation Mendelian randomization analysis.

All of the statistical analyses were carried out using the R program (version 4.2.1) and the package TwoSampleMR, and all *P* values were two-tailed^20^.

## Results

Among the 98232 included participants, there were 9928 with urolithiasis and 26217 who suffered from MAFLD. The prevalence of urolithiasis and MAFLD was 10.1% and 26.7%, respectively, and the inspection method was based on ultrasonic imaging. The mean age of the participants was 44.08 ± 12.63 years, with a male proportion of 56.5%. Among the 26217 patients with MAFLD, 3488 also suffered from urolithiasis at the same time, with a prevalence of 13.3%, which was higher than the 10.1% prevalence in the general population, accounting for 35.1% of all urolithiasis patients. The clinical and laboratory characteristics of the included participants are shown in **Table 2**. Binomial logistic regression analysis showed that MAFLD was associated with an increased risk of urolithiasis [OR, 1.563 (95% CI, 1.495 - 1.633)]. Through multivariable analysis, the adjusted ORs for urolithiasis were 1.204 (95% CI, 1.146 - 1.265) in model 1 and 1.137 (95% CI, 1.079 - 1.199) in model 2 after adjusting for influencing factors of urolithiasis in the logistic regression model (**Table 3**). After controlling for other factors, the odds ratio (OR) between MAFLD and urolithiasis remained statistically significant (p < 0.001).

**Figure 2:**
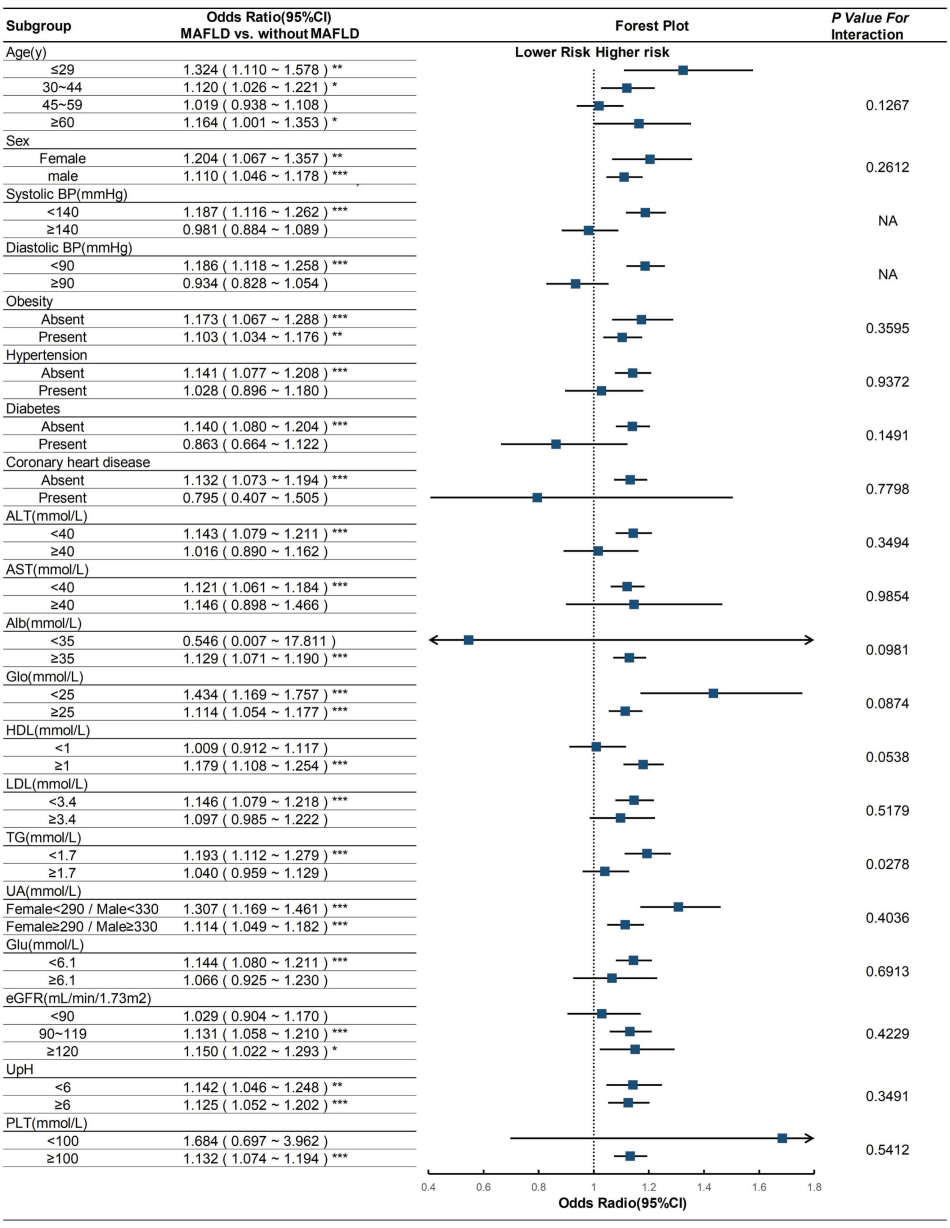
**Subgroup analyses on the OR of the risk of urolithiasis (MAFLD vs. without MAFLD).** Abbreviations: CI, confidence interval; BP, blood pressure; ALT, alanine aminotransferase; AST, aspartate aminotransferase; Alb, albumin; Glo, globulin; HDL, high-density lipoprotein cholesterol; LDL, low-density lipoprotein cholesterol; TG, triglycerides; UA, uric acid; Glu, fasting glucose; eGFR, estimated glomerular filtration rate; UpH, urine pH; PLT, platelet. Adjusted as model 2 (see Methods-Statistical Analyses section for descriptions of model 2). P for interaction was calculated by applying the Wald test. * *p*<0.05; ** p<0.01; *** *p*<0.001.

We subsequently performed a subgroup analysis to investigate the heterogeneity of the association between MAFLD and urolithiasis across different populations. The results revealed that this positive correlation persisted in most subgroups. There was no interaction between the subgroup factors, including age, sex, obesity, hypertension, diabetes, coronary heart disease, ALT, AST, Alb, Glo, HDL, LDL, UA, Glu, eGFR, UpH, and PLT, and MAFLD (**Figure 2**). Significant differences in the odds ratio (OR) of urolithiasis related to MAFLD were observed only in the high and low triglyceride (TG) groups, indicating an interaction between TG and MAFLD (p for interaction < 0.001).

**Figure 3:**
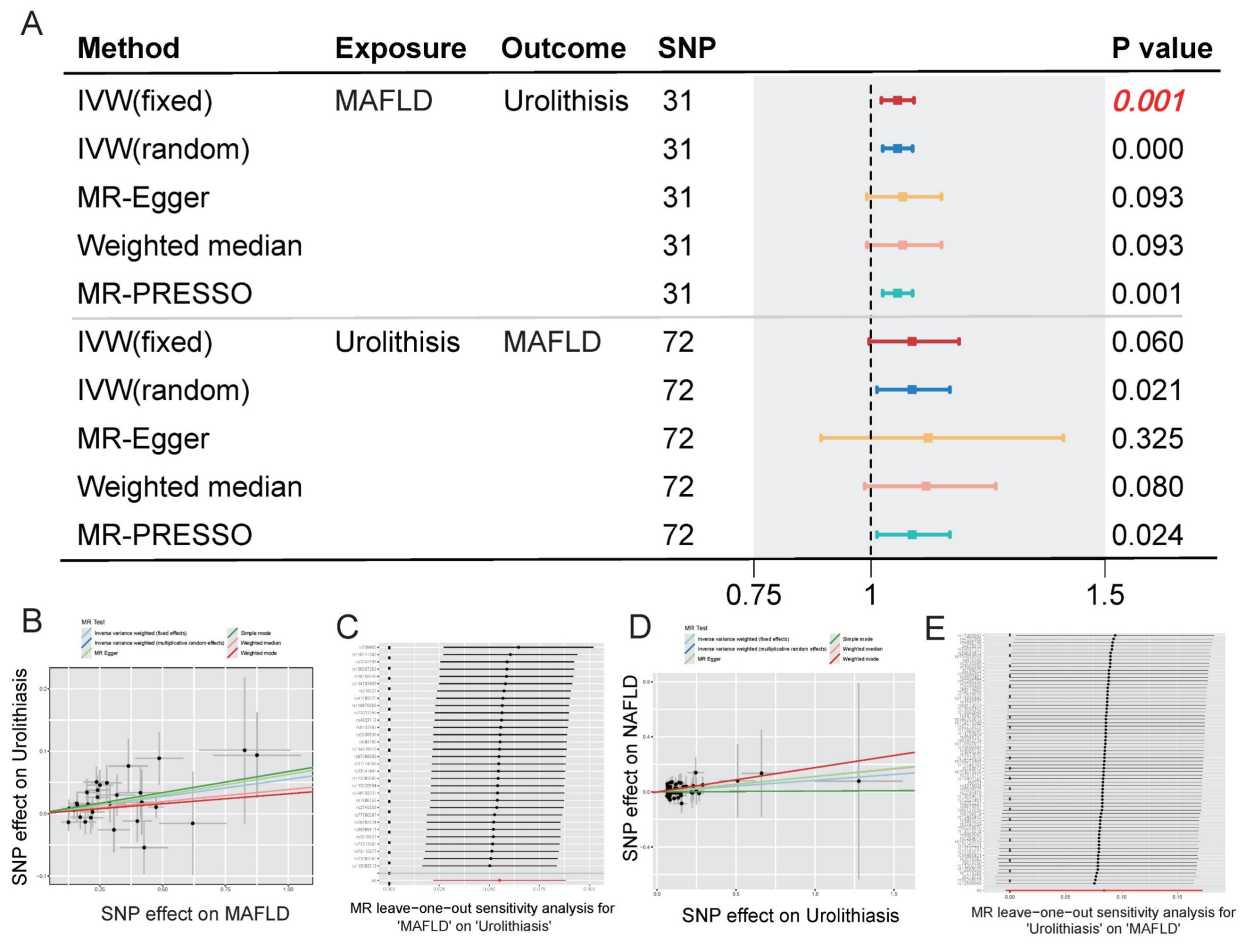
**Association between MAFLD and urolithiasis using bidirectional MR methods. (A)** Forest plot showing the estimated effects of exposure on outcome using various MR methods. The SNPs used are 31 for MAFLD and 72 for Urolithiasis. The methods include IVW (fixed and random effects), MR-Egger, Weighted median, and MR-PRESSO. The P values are provided for each method, with significant associations indicated in bold. **(B-C)** Scatter plot and MR leave-one-out sensitivity analysis for MAFLD on urolithiasis.** (D-E)** Scatter plot and MR leave-one-out sensitivity analysis for urolithiasis on MAFLD.

To eliminate the influence of confounding factors, we next analyzed the relationship between MAFLD and urolithiasis using genetic variables. **Supplementary Tables 1** and** 2** present the selected SNPs associated with genetically determined MAFLD and urolithiasis and their corresponding statistical measures, respectively. In the subsequent bidirectional Mendelian randomization analysis, the OR of urolithiasis impacted by genetically determined MAFLD was 1.5667 (95% CI: 1.0224 -1.0921, p = 0.001). The OR of MAFLD impacted by genetically determined urolithiasis was 1.0880 (95% CI: 0.9963 - 1.1882, p =0.06). **Figure 3A** presents a forest plot of the results from different MR methods, showing a causal association between genetically determined MAFLD and an increased risk of urolithiasis. In contrast, the association between urolithiasis and a higher risk of MAFLD was not statistically significant. The results remained highly consistent across the various Mendelian randomization methods, and the LOO analysis indicated that our conclusions were not driven by a small number of SNPs, further demonstrating the robustness of the findings (**Figure 3B-E**).

**Figure 4:**
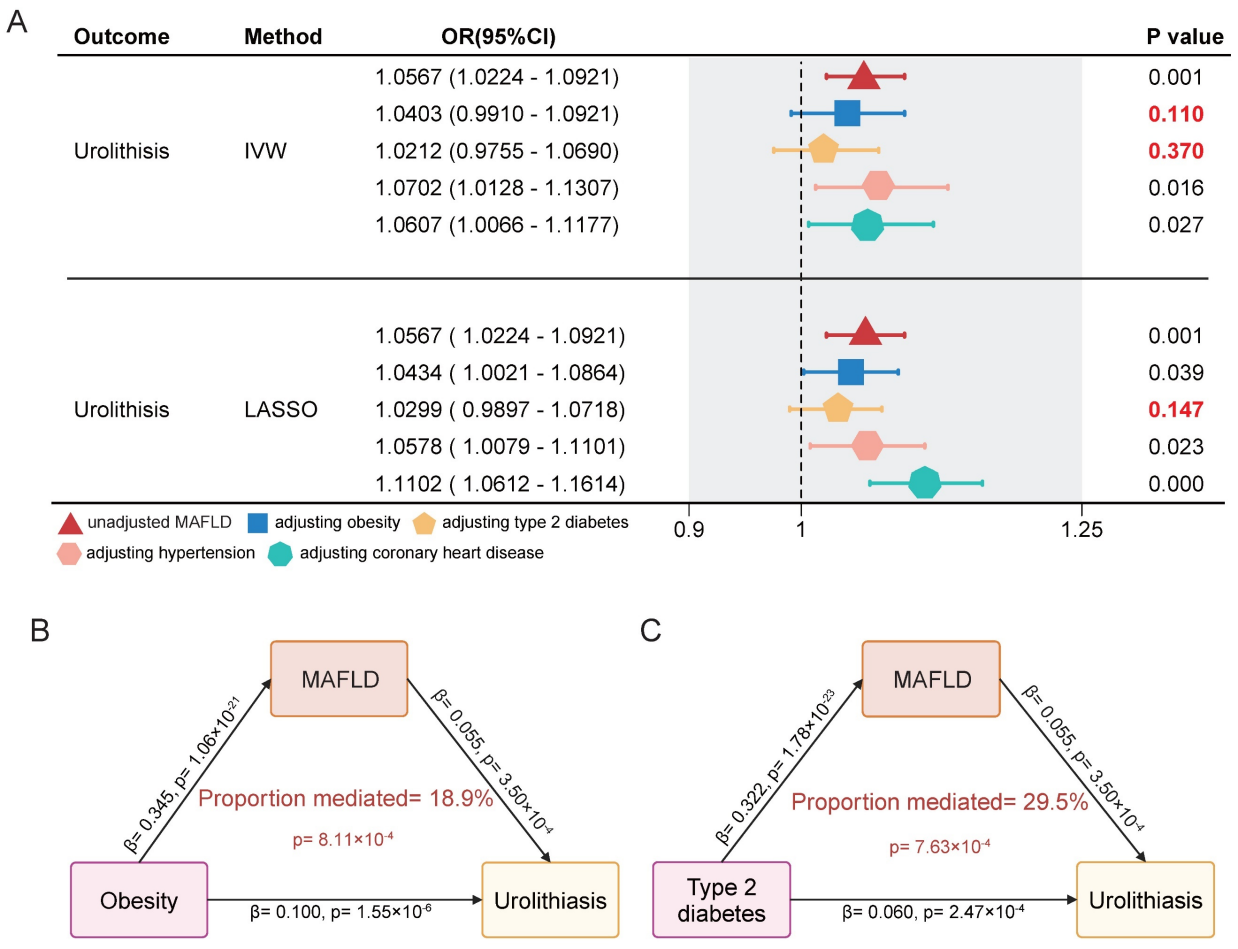
**Multivariable MR analysis and mediation analysis of the association between MAFLD and urolithiasis. (A)** The direct causal effect of MAFLD on urolithiasis by adjusting obesity, type2 diabetes, hypertension and coronary heart disease. The methods include IVW and LASSO. **(B-C)** Mediation analysis diagram showing the direct and indirect effects of obesity or type 2 diabetes on urolithiasis through MAFLD.

To account for the influence of conditions closely related to both MAFLD and urolithiasis, such as obesity and diabetes, we conducted a multivariable MR analysis. The results showed that the OR value for MAFLD in a univariable model significantly decreased after adjusting for obesity and diabetes, with the effect on urolithiasis losing significance under the IVW method. However, adjustments for hypertension and coronary heart disease did not lead to notable changes in the effect (**Figure 4A**). Through further mediation analysis, we found that MAFLD mediates the causal effect of obesity and type 2 diabetes on urolithiasis. MAFLD mediated 18.9% of the causal effect of obesity on urolithiasis and 29.5% of the causal effect of type 2 diabetes on urolithiasis (**Figure 4B-C**). Additionally, no evidence of horizontal pleiotropy was detected in any of the MR analyses (**Table 4**).

## Discussion

In this study, we systematically investigated the association between MAFLD and the risk of urolithiasis using cross-sectional data and explored their potential causal relationship at a genetic level. Our cross-sectional analysis and logistic regression indicated a significant positive correlation between MAFLD and urolithiasis, consistent with previous findings, such as those reported by Qin *et al.*^10^, ^13^. Moreover, our subgroup analysis revealed no significant interactions between MAFLD and most urolithiasis risk factors, with the exception of the triglyceride (TG) subgroup, where a significant difference in the effect of MAFLD on urolithiasis was observed between high and low TG levels. Additionally, bidirectional Mendelian randomization (MR) analysis suggested a causal relationship between MAFLD and an increased risk of urolithiasis, while no reverse causal relationship was detected. In multivariable Mendelian randomization, we adjusted for obesity, type 2 diabetes, hypertension, and coronary heart disease and found that the association between MAFLD and urolithiasis weakened or lost statistical significance after adjusting for obesity and type 2 diabetes. Subsequent mediation analysis further indicated that MAFLD mediates the effect of obesity and type 2 diabetes on the risk of urolithiasis. These findings indicate that MAFLD indeed increases the risk of urolithiasis directly or indirectly as a mediating factor in metabolic disorders, suggesting a causal relationship between the two conditions.

We observed an interaction between MAFLD and triglyceride levels in the subgroup analysis. Specifically, the impact of MAFLD on kidney stones was not significant in the high triglyceride group (≥1.7 mmol/L) but was significant in the low triglyceride group (<1.7 mmol/L). We propose that elevated triglycerides may obscure the independent effects of MAFLD and might independently contribute to kidney stone risk, likely through metabolic disturbances such as insulin resistance, hyperuricemia, and metabolic syndrome. In the high triglyceride group, the effect of triglycerides might overshadow the additional contribution of MAFLD. In the low triglyceride group, the role of MAFLD might be more pronounced because these individuals have fewer other metabolic risk factors for kidney stones, making the impact of MAFLD more observable.

Previous studies examining the relationship between MAFLD and urolithiasis have primarily relied on cross-sectional designs, lacking detailed subgroup analyses of urolithiasis risk factors and generally reporting a universal positive association between MAFLD and urolithiasis risk^9^, ^21^. However, recent findings indicate that this association may vary across different populations. For instance, a cohort study in Korea found that MAFLD is associated with an increased incidence of kidney stones in men but not in women^22^. In contrast, the NHANES III study, focused mainly on U.S. adults, reported that MAFLD is linked to a higher risk of kidney stones in women but not in men^23^. It is probable that these discrepancies are the result of differences in metabolic factors and methodological approaches. For instance, the Korean cohort exhibited significantly elevated rates of obesity, diabetes, and hypertension among males, whereas the NHANES III study demonstrated a higher prevalence of these conditions among females. Both MAFLD and urolithiasis are closely associated with metabolic syndrome, with MAFLD acting as both a cause and a consequence of metabolic syndrome^24^, ^25^. Consequently, higher prevalence and potential association are more pronounced in populations with metabolic dysregulation, whereas such correlations are challenging to confirm in metabolically normal populations. Our findings support this as well, showing that MAFLD plays a mediating role in the risk of urolithiasis driven by obesity and type 2 diabetes. Combined with our genetic evidence, the causal relationship between MAFLD and urolithiasis becomes more readily identifiable.

A recent meta-analysis synthesizing findings from seven cross-sectional studies and one prospective cohort study reported a significant association between MAFLD and an increased risk of urolithiasis^26^. However, the prospective cohort study within this meta-analysis showed no such association. Notably, this cohort study is the same Korean cohort mentioned earlier in our paper, and given the inclusion population issues and result variability in individual studies, we have reason to believe that its conclusions are unreliable. Similarly, a recent two-sample MR study concluded that MAFLD and urolithiasis were not associated^26^. Although this study used biopsy-diagnosed NAFLD data, its MAFLD information was derived from the Million Veteran Program (MVP) cohort, which predominantly includes European, African, and Asian American populations. In contrast, the urolithiasis outcome data was sourced from UK Biobank and FinnGen, both of which are predominantly European cohorts. The significant methodological differences and genetic analyses conducted on varied populations likely introduce substantial bias, which may explain the discrepancy between their findings and ours.

MAFLD and urolithiasis share several common risk factors, including obesity, diabetes, hypertension, and metabolic syndrome^27^, ^28^. Some of these shared risk factors may directly impact the risk of urolithiasis and also influence it indirectly via MAFLD, particularly obesity and type 2 diabetes. Multiple previous studies have shown that obesity and insulin resistance are important pathogenic factors of MAFLD^29^, ^30^. In addition, insulin resistance can also increase the risk of calcium stone formation by reducing the excretion of uric citrate and contribute to low uric ammonium and low pH which can elevate the risk of uric acid precipitation^31^-^33^. Moreover, an independent association exists between hypertension and the occurrence of urolithiasis, which may be caused by increased urinary calcium excretion in patients with hypertension^34^. Prior studies have demonstrated that a rise in SBP in the normal range is associated with noticeably increased chances of MAFLD, independent of other confounding variables^35^. These suggest that hypertension may not influence urolithiasis through MAFLD, aligning with our study's findings. Coronary heart disease is mainly the common development consequence of these two diseases^36^, ^37^. It is important to focus on these common risk factors and assess the risk of urolithiasis in patients diagnosed with MAFLD. Researchers have discovered that xanthine oxidase (XO), a rate-limiting enzyme that catalyzes the synthesis of uric acid, is crucial in the connection between MAFLD and hyperuricemia^38^. Meanwhile, it is well-known that hyperuricemia is an important risk factor for urolithiasis^39^, ^40^. Consequently, XO may represent a novel therapeutic target for these diseases, and further research into shared regulatory mechanisms could identify additional treatment targets.

We acknowledge that this study has some limitations. First, in the cross-sectional study, MAFLD and urolithiasis were diagnosed through ultrasonography, a method often employed in large population studies. However, ultrasonography is a subjective technique with lower sensitivity and specificity for these two diseases. More accurate diagnostic tools, such as liver biopsy and computed tomography, should be used. Therefore, additional studies utilizing computed tomography are required to validate these findings, as it offers more precise diagnoses and allows for classification of disease severity. Second, there was no prior history of urolithiasis or information on stone composition. To compensate for the absence of knowledge on stone composition, we altered the UpH to represent varied stone kinds and urine chemistry. MAFLD may not be causally associated with all types of urinary stones, which could account for some inconsistency in epidemiological findings; however, without specific stone composition data, we were unable to stratify participants by stone type. Third, our cross-sectional analysis relied on single-center medical examination information. We expect further multicenter prospective studies to validate the causal relationship between MAFLD and urolithiasis. Lastly, due to limited MAFLD and urolithiasis data from unified methodological sources, our MR analysis was conducted primarily in European populations, and further validation in diverse populations with expanded GWAS data is warranted.

## Conclusions

This study provides robust evidence that MAFLD contributes causally to an increased risk of urolithiasis, as shown through both cross-sectional analyses and Mendelian Randomization (MR). The findings underscore MAFLD not only as a significant risk factor for urolithiasis but also as a mediator in the pathway from obesity and type 2 diabetes to urolithiasis, highlighting complex interconnections between metabolic disorders and kidney stone formation. The identified causal association between MAFLD and urolithiasis suggests potential opportunities for early intervention and targeted prevention strategies in individuals at risk for both conditions. Further research is warranted to refine our understanding of MAFLD's specific mechanisms in urolithiasis development, which could guide innovative therapeutic approaches aimed at modifying metabolic pathways to reduce the burden of kidney stones.

## Supplementary Material

Supplementary tables.

## Figures and Tables

**Table 1:** Characteristics of GWAS enrolled in the MR study.

Items	GWAS ID	Sample size	Cases	Controls	Consortium
MAFLD	finn-b-NAFLD	412181	2568	409613	FinnGen
Urolithiasis	finn-b-N14_CALCUKIDUR	411237	10556	400681	FinnGen
Obesity	finn-b-E4_OBESITY	412055	23971	388084	FinnGen
Type 2 diabetes	finn-b-T2D	400197	65085	335112	FinnGen
Hypertension	finn-b-I9_HYPTENS	412113	122996	289117	FinnGen
CHD	finn-b-I9_CHD	412181	46959	365222	FinnGen

GWAS: genome-wide association study; MR: mendelian randomization; MAFLD, metabolic dysfunction-associated fatty liver disease; NAFLD: nonalcoholic fatty liver disease; T2D: Type 2 diabetes; CHD: coronary heart disease.

**Table 2:** Basic characteristics of included participants with or without urolithiasis.

	All Participants	Participants without	Participants with	*P*-value
Variables	(n=98232)	Urolithiasis (n=88304)	Urolithiasis (n=9928)	
Urolithiasis^a^ present (%)	9928 (10.1)	0 (0.0)	9928 (100.0)	<0.001
Age, y	41.22 ± 12.95	40.90 ± 12.94	44.08 ± 12.63	<0.001
Sex = male (%)	55470 (56.5)	48354 (54.8)	7116 (71.7)	<0.001
Obesity present (%)	41972 (42.7)	36842 (41.7)	5130 (51.7)	<0.001
HBP present (%)	8090 (8.2)	6838 (7.7)	1252 (12.6)	<0.001
DM present (%)	2349 (2.4)	2011 (2.3)	338 (3.4)	<0.001
CHD present (%)	529 (0.5)	457 (0.5)	72 (0.7)	0.009
MAFLD present (%)	26217 (26.7)	22729 (25.7)	3488 (35.1)	<0.001
BMI^b^, kg/m^2^	23.56 ± 3.37	23.48 ± 3.37	24.27 ± 3.28	<0.001
SBP, mmHg	123.85 ± 17.92	123.48 ± 17.81	127.18 ± 18.54	<0.001
DBP, mmHg	75.79 ± 12.02	75.52 ± 11.94	78.24 ± 12.49	<0.001
ALT, U/L	23.32 ± 22.19	23.08 ± 22.37	25.46 ± 20.39	<0.001
AST, U/L	21.95 ± 12.47	21.86 ± 12.62	22.80 ± 10.97	<0.001
TP, g/L	76.01 ± 3.96	76.03 ± 3.96	75.88 ± 3.98	<0.001
Alb, g/L	46.11 ± 2.60	46.11 ± 2.59	46.07 ± 2.62	0.132
Glo, g/L	29.91 ± 3.56	29.92 ± 3.56	29.82 ± 3.56	0.007
GGT, U/L	31.09 ± 35.20	30.52 ± 34.78	36.14 ± 38.36	<0.001
TBIL, μmol/L	13.64 ± 5.44	13.61 ± 5.46	13.94 ± 5.28	<0.001
IBIL, μmol/L	9.97 ±4.20	9.94 ±4.23	10.21 ±3.93	<0.001
DBIL, μmol/L	3.68 ±1.72	3.67 ±1.74	3.73 ±1.57	0.002
TC, mmol/L	4.53 ± 0.87	4.52 ± 0.87	4.62 ± 0.89	<0.001
HDL, mmol/L	1.28 ± 0.31	1.29 ± 0.31	1.23 ± 0.29	<0.001
LDL, mmol/L	2.73 ± 0.75	2.72 ± 0.74	2.81 ± 0.77	<0.001
TG, mmol/L	1.47 ± 1.28	1.44 ± 1.26	1.67 ± 1.47	<0.001
eGFR^c^, mL/min/1.73m^2^	112.02 ± 17.23	112.50 ± 17.10	107.72 ± 17.77	<0.001
UA, mg/dL	342.17 ± 95.51	339.27 ± 94.32	367.94 ± 101.97	<0.001
Glu, mmol/L	5.32 ± 1.11	5.30 ± 1.09	5.46 ± 1.26	<0.001
PLT, (×10^9^/L)	228.48 ± 56.30	228.69 ± 56.36	226.65 ± 55.70	0.001
UpH	6.12 ± 0.65	6.12 ± 0.65	6.09 ± 0.64	<0.001

^a^ Regardless of size, structures seen by an ultrasonography test were considered to be kidney stones. ^b^ Calculated by dividing the weight in kilograms by the square of the height in meters. ^c^ derived from the CKD-EPI equation (details can be found in the Methods section). HBP: high blood pressure; DM: diabetes mellitus; CHD: coronary heart disease; MAFLD, metabolic dysfunction-associated fatty liver disease; BMI: body mass index; SBP: systolic blood pressure; DBP: diastolic blood pressure; ALT: alanine aminotransferase; AST: aspartate aminotransferase; TP: total protein; Alb: albumin; Glo: globulin; GGT: γ-glutamyl transpeptidase; TBIL: total bilirubin; IBIL: indirect bilirubin; DBIL: direct bilirubin; TC: total cholesterol; HDL: high-density lipoprotein cholesterol; LDL: low-density lipoprotein cholesterol; TG: triglyceride; eGFR: estimated glomerular filtration rate; UA: uric acid; Glu: fasting glucose; PLT: platelet; UpH: urine pH

**Table 3:** Association between MAFLD and urolithiasis using an extended model approach

	Odds ratio of MAFLD	*P-*value
Unadjusted	1.563 (1.495 ~ 1.633)	<0.001
Model 1^a^	1.204 (1.146 ~ 1.265)	<0.001
Model 2^b^	1.137 (1.079 ~ 1.199)	<0.001

^a^ Model 1: Adjusted for age, sex, obesity, HBP, DM, and CHD. ^b^ Model 2: Model 1 plus, ALT, AST, Alb, Glo, HDL, LDL, TG, UA, Glu, eGFR, UPH, and PLT. (See Methods-Statistical analyses section for descriptions of models 1 and 2).

**Table 4:** Sensitivity analysis of adjusted MAFLD with risk of urolithiasis in multivariable MR analyses.

	Heterogeneity			Pleiotropy	
Exposure	Cochrane's Q	*P*		Interccept	*P*
MAFLD adjusted by obesity	261	3.89E-05		-5.57E-05	0.990
MAFLD adjusted by T2D	532	3.54E-09		7.30E-03	0.300
MAFLD adjusted by hypertension	589	4.05E-12		-2.68E-03	0.091
MAFLD adjusted by CHD	297	1.11E-07		4.00E-03	0.306

^a^ Multivariable MR-Egger was applied to assess pleiotropy, with no evidence of pleiotropy found in any analysis.

## Abbreviations

MAFLD: metabolic dysfunction-associated fatty liver disease
MR: Mendelian randomization
HBP: high blood pressure
DM: diabetes mellitus
CHD: coronary heart disease
NAFLD: nonalcoholic fatty liver disease
BMI: body mass index
SBP: systolic blood pressure
DBP: diastolic blood pressure
ALT: alanine aminotransferase
AST: aspartate aminotransferase
TP: total protein
Alb: albumin
Glo: globulin
GGT: γ-glutamyl transpeptidase
TBIL: total bilirubin
IBIL: indirect bilirubin
DBIL: direct bilirubin
TC: total cholesterol
HDL: high-density lipoprotein cholesterol
LDL: low-density lipoprotein cholesterol
TG: triglycerides
eGFR: estimated glomerular filtration rate
UA: uric acid
Glu: fasting glucose
PLT: platelet
UpH: urine pH. OR, odds ratio
CI: confidence interval. EA, effect allele
NEA: noneffect allele
EAF: effect allele frequency
SNP: single-nucleotide polymorphism. FinnGen, FinnGen Consortium
UKBB: UK Biobank
