# Supplementary material for: Metabolic dysfunction-associated fatty liver disease as a mediator of urolithiasis risk: evidence from cross-sectional and genetic studies
Source: Int J Med Sci. 2025 Jan 21;22(4):764–74. doi: 10.7150/ijms.106824 (PMC11843138; doi:10.7150/ijms.106824)
Supplement: Supplementary file 1 — Supplementary tables. [file ijmsv22p0764s1.pdf]

Supplementary Table 1. Single-Nucleotide Polymorphisms used as instrumental variables for MAFLD.

						Steiger Test		Exposure		Outcome	
								MAFLD		Urolithiasis	
**SNP**	**EA**	**NEA**	**EAF**	**R^2**	**F**	**direction**	**P**	**Beta**	**P**	**Beta**	**P**
rs10158849	A	G	0.152	5.44E-05	22	TRUE	0.004	-0.192	2.36E-06	0.013	0.504
rs11174359	A	G	0.117	4.84E-05	20	TRUE	0.008	-0.203	8.41E-06	-0.015	0.491
rs113092519	C	G	0.034	6.31E-05	26	TRUE	0.035	-0.486	3.68E-07	-0.089	0.036
rs113228594	T	A	0.003	5.09E-05	21	TRUE	0.009	0.826	4.92E-06	0.102	0.385
rs114737456	G	A	0.041	5.56E-05	23	TRUE	0.004	0.306	1.84E-06	-0.026	0.471
rs114916678	C	T	0.060	5.02E-05	21	TRUE	0.005	-0.290	5.80E-06	-0.016	0.597
rs13141441	A	G	0.227	5.41E-05	22	TRUE	0.005	-0.161	2.50E-06	-0.012	0.458
rs140141593	A	C	0.031	5.01E-05	21	TRUE	0.021	-0.427	5.93E-06	0.054	0.200
rs142082590	T	C	0.031	4.81E-05	20	TRUE	0.010	-0.412	9.03E-06	-0.034	0.413
rs146132021	C	G	0.050	4.83E-05	20	TRUE	0.012	-0.318	8.63E-06	-0.030	0.365
rs149476868	T	C	0.007	5.26E-05	22	TRUE	0.002	0.620	3.47E-06	-0.016	0.851
rs150057262	G	C	0.050	1.17E-04	48	TRUE	0.000	0.399	4.51E-12	-0.012	0.722
rs1806155	T	G	0.199	5.34E-05	22	TRUE	0.008	0.158	2.91E-06	0.016	0.352
rs219227	T	C	0.826	4.94E-05	20	TRUE	0.003	0.172	6.86E-06	-0.006	0.762
rs2238538	A	G	0.177	4.85E-05	20	TRUE	0.004	0.160	8.32E-06	0.007	0.719
rs2518827	G	A	0.899	5.82E-05	24	TRUE	0.020	0.242	1.04E-06	0.038	0.109
rs2745359	C	T	0.083	6.22E-05	25	TRUE	0.004	0.241	4.43E-07	0.026	0.302
rs28703824	A	G	0.127	6.02E-05	25	TRUE	0.017	0.199	6.77E-07	0.034	0.107
rs35685817	A	G	0.028	5.44E-05	22	TRUE	0.035	0.364	2.35E-06	0.076	0.081
rs41293070	T	C	0.083	4.91E-05	20	TRUE	0.003	0.214	7.24E-06	-0.006	0.805
rs4823112	T	C	0.091	5.71E-05	23	TRUE	0.001	0.221	1.33E-06	0.003	0.906
rs67088699	T	C	0.408	4.98E-05	20	TRUE	0.005	0.128	6.22E-06	0.009	0.553
rs693190	G	T	0.019	5.19E-05	21	TRUE	0.003	0.417	4.05E-06	0.018	0.726

rs7043196	T	C	0.476	4.88E-05	20	TRUE	0.013	-0.125	7.82E-06	0.014	0.336
rs72893301	A	G	0.094	5.24E-05	21	TRUE	0.072	-0.237	3.59E-06	-0.051	0.036
rs73027058	G	C	0.109	5.27E-05	22	TRUE	0.001	-0.221	3.38E-06	-0.003	0.909
rs73115877	C	T	0.091	5.70E-05	23	TRUE	0.036	-0.251	1.36E-06	-0.046	0.062
rs738409	G	C	0.227	6.06E-04	249	TRUE	0.000	0.474	5.51E-56	0.011	0.530
rs77700287	T	C	0.011	6.12E-05	25	TRUE	0.010	-0.876	5.51E-07	-0.094	0.170
rs77917801	T	G	0.067	5.21E-05	21	TRUE	0.042	-0.278	3.87E-06	-0.049	0.080
rs8137962	G	A	0.489	6.82E-05	28	TRUE	0.001	0.148	1.25E-07	0.006	0.683

Abbreviations: EA, effect allele; NEA, noneffect allele; EAF, effect allele frequency; SNP, single-nucleotide polymorphism.

Supplementary Table 2. Single-Nucleotide Polymorphisms used as instrumental variables for urolithiasis.

						Steiger Test		Exposure		Outcome	
								Urolithiasis		MAFLD	
SNP	EA	NEA	EAF	R^2	F	direction	P	Beta	P	Beta	P
rs1061124	C	T	0.052	4.87E-05	20	TRUE	1.77E-03	0.138	7.60E-06	0.004	0.954
rs10992648	G	A	0.381	5.40E-05	22	TRUE	1.59E-03	0.068	2.47E-06	-0.007	0.805
rs11082442	G	C	0.265	6.08E-05	25	TRUE	7.12E-04	-0.080	5.74E-07	0.007	0.829
rs111891490	T	C	0.091	9.60E-05	39	TRUE	1.65E-05	0.149	3.34E-10	-0.010	0.846
rs11202736	T	A	0.347	4.85E-05	20	TRUE	3.16E-02	0.066	7.88E-06	0.042	0.152
rs114111299	C	G	0.128	4.94E-05	20	TRUE	2.10E-03	0.093	6.57E-06	-0.007	0.873
rs114502917	T	C	0.017	4.80E-05	20	TRUE	1.97E-03	0.223	8.92E-06	-0.007	0.947
rs11750874	C	A	0.881	8.31E-05	34	TRUE	3.59E-05	-0.123	5.01E-09	0.000	0.995
rs12080020	T	C	0.478	5.56E-05	23	TRUE	1.53E-02	-0.067	1.73E-06	-0.038	0.175
rs12402142	T	A	0.018	5.09E-05	21	TRUE	3.15E-03	0.229	4.73E-06	0.044	0.687
rs12485415	T	G	0.043	4.86E-05	20	TRUE	1.25E-02	0.150	7.74E-06	0.065	0.345
rs12585865	C	T	0.410	5.03E-05	21	TRUE	7.94E-02	0.065	5.42E-06	0.059	0.038
rs12723578	A	G	0.214	5.30E-05	22	TRUE	1.38E-03	0.079	3.03E-06	0.005	0.883
rs12921916	C	T	0.264	4.88E-05	20	TRUE	4.56E-03	0.071	7.53E-06	-0.015	0.639
rs13059018	C	G	0.103	6.50E-05	27	TRUE	9.93E-04	-0.125	2.33E-07	-0.024	0.605
rs13127707	G	A	0.327	1.41E-04	58	TRUE	2.16E-06	0.114	2.63E-14	0.028	0.357
rs13148163	C	T	0.572	5.29E-05	22	TRUE	1.83E-03	-0.066	3.11E-06	-0.007	0.795
rs13213218	G	A	0.220	5.21E-05	21	TRUE	1.26E-02	0.077	3.70E-06	0.037	0.271
rs1376001	T	C	0.436	5.96E-05	25	TRUE	1.50E-03	0.070	7.40E-07	0.013	0.642
rs1411992	C	G	0.837	5.13E-05	21	TRUE	1.65E-03	0.089	4.37E-06	-0.005	0.885
rs146253613	G	T	0.036	6.84E-05	28	TRUE	1.89E-04	-0.217	1.13E-07	0.002	0.978
rs147750540	A	G	0.061	2.65E-04	109	TRUE	1.16E-11	0.289	1.50E-25	0.051	0.390
rs149273486	C	G	0.000	5.11E-05	21	TRUE	1.55E-03	1.277	4.59E-06	0.078	0.913

rs1497075	T	C	0.328	5.10E-05	21	TRUE	1.01E-02	0.068	4.70E-06	0.028	0.345
rs150350500	C	A	0.065	1.80E-04	74	TRUE	2.71E-09	-0.266	8.09E-18	0.011	0.849
rs151075016	T	C	0.003	4.89E-05	20	TRUE	3.08E-03	0.509	7.24E-06	0.081	0.761
rs1546737	T	C	0.367	7.19E-05	30	TRUE	2.49E-04	0.079	5.44E-08	-0.008	0.796
rs17486892	C	T	0.241	5.78E-05	24	TRUE	2.64E-02	-0.082	1.10E-06	0.057	0.082
rs181673	C	A	0.511	5.61E-05	23	TRUE	1.33E-03	0.068	1.57E-06	0.008	0.790
rs1866399	A	G	0.225	4.78E-05	20	TRUE	1.36E-02	-0.076	9.30E-06	0.032	0.344
rs1970645	A	G	0.432	5.44E-05	22	TRUE	1.13E-03	-0.068	2.23E-06	-0.004	0.898
rs2038395	A	G	0.507	5.31E-05	22	TRUE	7.97E-03	0.067	2.97E-06	-0.026	0.356
rs2079742	C	T	0.178	1.80E-04	74	TRUE	6.43E-08	-0.165	8.33E-18	-0.035	0.340
rs219773	A	G	0.215	2.16E-04	89	TRUE	4.04E-09	-0.166	3.89E-21	-0.038	0.262
rs2332237	A	C	0.693	6.34E-05	26	TRUE	1.23E-03	-0.077	3.26E-07	-0.016	0.589
rs2744475	G	C	0.345	8.43E-05	35	TRUE	1.36E-04	0.087	3.93E-09	0.015	0.620
rs34488421	C	A	0.304	6.98E-05	29	TRUE	3.40E-03	0.083	8.44E-08	0.038	0.223
rs3742239	G	A	0.290	5.40E-05	22	TRUE	8.11E-03	0.073	2.47E-06	0.030	0.332
rs3788338	G	A	0.800	5.19E-05	21	TRUE	4.59E-03	-0.080	3.80E-06	0.022	0.538
rs415745	T	C	0.478	4.79E-05	20	TRUE	1.62E-02	0.062	9.10E-06	-0.029	0.299
rs4498196	C	A	0.381	6.89E-05	28	TRUE	3.13E-03	-0.078	1.01E-07	0.033	0.251
rs4551	C	G	0.002	6.66E-05	27	TRUE	6.74E-04	0.661	1.66E-07	0.135	0.668
rs4782366	C	T	0.432	6.29E-05	26	TRUE	9.26E-03	0.072	3.69E-07	-0.040	0.159
rs5009225	T	C	0.692	6.87E-05	28	TRUE	6.46E-04	-0.080	1.07E-07	-0.015	0.623
rs56208978	G	C	0.194	5.25E-05	22	TRUE	4.94E-02	0.082	3.41E-06	0.067	0.062
rs5756819	C	A	0.617	5.79E-05	24	TRUE	3.55E-03	-0.070	1.06E-06	-0.022	0.447
rs57719175	A	G	0.390	7.13E-05	29	TRUE	1.40E-04	-0.079	6.12E-08	0.001	0.974
rs59040412	T	C	0.100	4.88E-05	20	TRUE	1.43E-02	-0.108	7.53E-06	0.048	0.309
rs59112864	A	T	0.331	4.95E-05	20	TRUE	4.35E-03	0.067	6.45E-06	-0.014	0.631
rs60258676	C	T	0.221	5.35E-05	22	TRUE	8.64E-03	0.079	2.72E-06	0.033	0.327
rs610758	C	T	0.558	7.75E-05	32	TRUE	1.68E-03	-0.080	1.64E-08	-0.034	0.227
rs62286255	T	C	0.211	5.07E-05	21	TRUE	5.99E-03	0.078	5.00E-06	-0.024	0.496
rs62472728	T	C	0.092	4.95E-05	20	TRUE	1.66E-03	0.107	6.47E-06	-0.003	0.948

rs66512073	T	C	0.365	5.97E-05	25	TRUE	9.97E-04	0.072	7.28E-07	-0.009	0.763
rs68137036	G	A	0.306	6.14E-05	25	TRUE	1.75E-03	0.076	5.08E-07	0.018	0.549
rs7259073	T	C	0.904	5.37E-05	22	TRUE	1.16E-02	0.117	2.59E-06	0.055	0.257
rs72927451	T	C	0.040	5.34E-05	22	TRUE	3.29E-03	-0.184	2.78E-06	-0.040	0.595
rs73163577	A	G	0.016	5.48E-05	23	TRUE	1.34E-02	0.245	2.06E-06	0.140	0.210
rs74087222	A	C	0.046	4.79E-05	20	TRUE	2.42E-02	-0.155	9.00E-06	0.084	0.209
rs76599394	G	A	0.020	7.10E-05	29	TRUE	1.64E-04	0.248	6.59E-08	0.007	0.941
rs7719168	C	A	0.122	4.80E-05	20	TRUE	4.89E-02	0.094	8.91E-06	0.071	0.097
rs77663766	A	C	0.085	5.49E-05	23	TRUE	1.03E-03	0.116	2.00E-06	-0.006	0.911
rs7811777	G	A	0.439	5.11E-05	21	TRUE	2.58E-03	0.065	4.52E-06	0.009	0.744
rs78250221	A	G	0.062	5.09E-05	21	TRUE	1.58E-02	-0.139	4.77E-06	-0.068	0.244
rs78884357	T	G	0.074	7.07E-05	29	TRUE	5.10E-04	-0.152	6.97E-08	0.026	0.631
rs796075	G	A	0.649	5.23E-05	22	TRUE	5.35E-03	-0.068	3.50E-06	0.021	0.482
rs925813	C	T	0.111	7.86E-05	32	TRUE	7.67E-04	-0.133	1.29E-08	0.042	0.351
rs9273368	A	G	0.277	6.80E-05	28	TRUE	4.77E-04	-0.084	1.25E-07	0.011	0.727
rs9483085	T	C	0.230	5.21E-05	21	TRUE	1.35E-02	-0.078	3.68E-06	-0.038	0.255
rs9902482	T	C	0.536	7.49E-05	31	TRUE	3.32E-04	-0.078	2.88E-08	0.013	0.634
rs9948737	A	G	0.062	5.25E-05	22	TRUE	1.30E-03	0.131	3.40E-06	-0.006	0.920
rs9970557	T	C	0.354	4.75E-05	20	TRUE	6.41E-03	-0.066	9.93E-06	-0.017	0.571

Abbreviations: EA, effect allele; NEA, noneffect allele; EAF, effect allele frequency; SNP, single-nucleotide polymorphism.
